# A serum biomarker panel for early detection of treatment-related cardiotoxicity in early HER2-positive breast cancer patients

**DOI:** 10.1097/MS9.0000000000004191

**Published:** 2025-10-27

**Authors:** Nadeem A Ahmed, Hatem H Abbas, Ward A Hasan, Faisal N Redwan, Ahmed A Ahmed, Zuhair A Al-Shehabi

**Affiliations:** a Department of Cardiovascular Diseases, Faculty of Medicine, Tishreen University Hospital, Latakia, Syria; b Cancer Research Center, Tishreen University Hospital, Latakia, Syria; c Faculty of Medicine, Tishreen University Hospital, Latakia, Syria; d Department of Laboratory and Clinical Biochemistry, Faculty of Medicine, Tishreen University Hospital, Latakia, Syria; e Department of Statistics and Programming, Faculty of Economics, Tishreen University, Latakia, Syria; f Department of Pathology, Faculty of Medicine, Tishreen University Hospital, Latakia, Syria

**Keywords:** cardiotoxicity, CRP, HER2+ breast cancer, NTproBNP, troponin I

## Abstract

**Background:**

Standard treatment for human epidermal growth factor 2 positive (HER2+) breast cancer poses a high risk of cardiotoxicity, however, it still lacks predictive biomarkers. This study assesses five candidate biomarkers for early treatment-related cardiotoxicity prediction.

**Methods:**

All enrolled patients received the full treatment protocol consisting of anthracycline followed by 12 months of trastuzumab alone or with pertuzumab. Patients were prospectively followed for 27 months. Measurements of high-sensitivity troponin I (hs-Tn I), high-sensitivity C reactive protein (hs-CRP), N-terminal pro b-type natriuretic peptide, interleukin-6, and uric acid were performed at baseline, after anthracyclines, and after four cycles of anti-HER2 agents. Left ventricular ejection fraction (LVEF) measurements and full cardiac examination were performed every 3 months from baseline point until study end. Cardiotoxicity was defined as either an absolute decrease in LVEF of ≥15% or a drop in LVEF of ≥10% from the baseline to <50%.

**Results:**

Among 44 patients, cardiotoxicity occurred in 11 patients (25%). Higher risk of cardiotoxicity was associated with hs-Tn I levels ≥82 ng/L measured after four cycles of anti-HER2 agents. Elevated hs-CRP values ≥2.8 mg/L after 4 cycles of anthracyclines were also associated with increased risk. The highest risk was observed when both elevated hs-Tn I (≥82 ng/L after four cycles of anti-HER2 agents) and elevated hs-CRP (after four cycles of anthracyclines) were present.

**Conclusion:**

The interaction between both hs-Tn I and hs-CRP demonstrates significant predictive value for cardiotoxicity risk related to HER2+ breast cancer treatment.

## Introduction

Human epidermal growth factor 2 positive (HER2+) breast cancer accounts for approximately 20% of breast malignancies, characterized by amplification of the HER2 gene and overexpression of its transmembrane tyrosine kinase receptor. While HER2 plays essential roles in normal tissue growth and development, its pathological overexpression drives tumor progression and correlates with poor prognosis^[1]^. Trastuzumab (TRAS), the first approved HER2-targeted monoclonal antibody, binds extracellular domains to inhibit oncogenic signaling pathways^[2,3]^.

**HIGHLIGHTS**

- Trastuzumab combined with anthracyclines increases cardiac dysfunction risk in human epidermal growth factor 2 positive (HER2+) patients.
- Trastuzumab blocks NRG1/ErbB signaling, intensifying anthracycline-induced cardiotoxicity.
- Left ventricular ejection fraction and cardiac imaging lack sensitivity for detecting early cardiotoxicity.
- Combined chemotherapy complicates biomarker analysis, requiring precise timing and choice.
- Early high-sensitivity troponin I and high-sensitivity C reactive protein levels may predict cardiotoxicity post HER2+ cancer treatment.

Despite its therapeutic benefits, TRAS induces cardiac dysfunction in 3–7% of patients during monotherapy, with incidence rising to 27% when combined with anthracyclines (ANT)^[3,4]^. This cardiotoxicity spectrum ranges from subclinical myocardial injury to symptomatic heart failure, emphasizing the critical need for predictive strategies to identify high-risk patients before clinical manifestations occur^[3]^.

Currently, no validated biomarkers predict this cancer therapy-related cardiotoxicity. This study evaluates five candidate biomarkers high-sensitivity troponin I (hs-Tn I), high-sensitivity C reactive protein (hs-CRP), interleukin 6 (IL-6), uric acid (U.A), and N-terminal pro b-type natriuretic peptide (NTproBNP) measured during the treatment of early HER2+ breast cancer, which refers to non-metastatic (M0) breast cancer according to National Comprehensive Cancer Network NCCN, and American Society of Clinical Oncology ASCO. Our objective is to assess the predictive value of these biomarkers for this cancer therapy-related cardiotoxicity development within 1-year post-treatment completion. This study employs the TITAN (Transparency In The reporting of Artificial INtelligence) checklist, developed by Agha *et al*^[5]^.

## Patients and methods

### Study population and study design

This study is considered a prospective cohort study, in which both laboratory investigators and echocardiographic examiners were blinded. All consecutive HER2+ breast cancer patients during 2022 were included, excluding patients with any history of cardiovascular diseases, diabetes mellitus, hyperlipidemia, kidney diseases, or stage IV breast cancer.

### Study protocol

**Figure 1.**
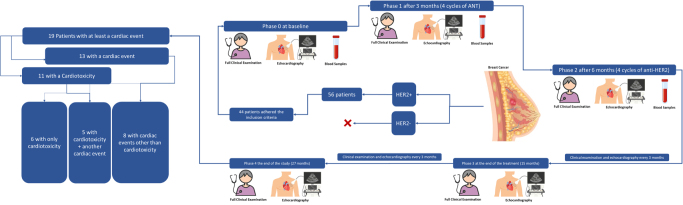
Study protocol and cardiac events. ANT, anthracyclines; HER2, human epidermal growth factor receptor 2.

All eligible patients underwent serial clinical evaluations including full echocardiographic exams and biomarker assays (hs-Tn I, hs-CRP, IL-6, U.A., and NTproBNP). Biomarker assessments were timed at three intervals: at baseline, post-ANT therapy (3 months after baseline), and after four cycles of anti-HER2 therapy (6 months after baseline). Clinical and echocardiographic follow-up continued throughout treatment and for 12 months post-therapy to detect cardiovascular complications (Fig. 1).

### Cardiotoxicity definition

Cardiotoxicity was defined by using left ventricular ejection fraction (LVEF) criteria aligned with cardio-oncology and heart failure-related guidelines and studies^[6–8]^. A symptomatic or asymptomatic LVEF decline ≥10% from baseline to <50%, or an absolute reduction ≥15% was classified as cardiotoxicity, ensuring compatibility with standard diagnostic thresholds.

### Primary and secondary endpoints

The primary endpoint was the occurrence of systolic dysfunction defined by either an LVEF decrease ≥15% or a ≥10% decline to <50%. Therapy was temporarily paused upon meeting these thresholds or the development of acute cardiac symptoms, with reassessment after 1–3 months to evaluate treatment continuation feasibility. The secondary endpoint of the study was the occurrence of any other cardiac event (anginal/ischemic event, development of hypertension, arrhythmia needed a pharmacological suppression).

### Imaging study

LVEF calculations were obtained using Simpson’s biplane method via apical four- and two-chamber views. All echocardiograms were performed using a standardized SIEMENS ACUSON X300 system. Multiple sonographers, blinded to patient data and study objectives, conducted imaging to minimize bias.

### Laboratory methods

Blood samples were processed via centrifugation (≥20 min) to isolate plasma. Hs-Tn I and hs-CRP levels were analyzed via enzyme-linked immunosorbent assay. All results underwent duplicate verification by independent, blind technicians.

### Statistical analysis

Data were analyzed using IBM SPSS Statistics 25.0. Logistic regression models assessed univariate and multivariate associations between biomarkers and cardiotoxicity. Receiver operating characteristic (ROC) curves evaluated biomarker diagnostic performance and cutoffs. Kaplan–Meier and Cox-regression were used to study the survival rates and correlations between biomarkers related to survival rates. Statistical significance was set at *P* < 0.05.

### Exposure to chemotherapy and anti-HER2 agents

All patients received ANT-based regimens: doxorubicin (60 mg/m^2^) or epirubicin (100 mg/m^2^) combined with cyclophosphamide (600 mg/m^2^) every 21 days for four cycles. Subsequent therapy included taxanes and HER2-targeted agents (TRAS: 8 mg/kg loading dose, then 6 mg/kg every 21 days for 1 year; pertuzumab (PERT): 840 mg loading dose, then 420 mg every 21 days if indicated).

### Study points definitions

We defined this study into five main phases: from phase 0, at baseline, to phase 4 at the end of the follow-up or the occurrence of cardiotoxicity (Fig. 1).

### Ethics committee approval

The authors declare that the study is fully compliant with the principles outlined in the World Medical Association Declaration of Helsinki: “Ethical Principles for Medical Research Involving Human Subjects.” Moreover, all participants in the study provided written informed consent.

## Results

### Study population

Of 56 patients diagnosed with HER2+ breast cancer, 44 met the inclusion criteria and completed the study. All patient characteristics are presented in Table 1.

**Table 1:** Patients’ characteristics

		Num	%	*P*-value of cardiotoxicity development risk
Type of therapy	Adjuvant	35	79.5	>0.05
	Neoadjuvant	9	20.5	
Position of tumor	Right	19	43.2	>0.05
	Left	25	56.8	
Stage	IA	4	9.1	>0.05
	IB	1	2.3	
	IIA	15	34.1	
	IIB	10	22.7	
	IIIA	10	22.7	
	IIIB	2	4.5	
	IIIC	2	4.5	
Hormonal receptors	ER +	17	38.6	>0.05
	PR +	15	34.1	0.042
Ki67	≤5%	3	6.8	>0.05
	6–29%	18	40.9	
	≥30	23	52.3	
Anthracyclines	Doxorubicin	32	72.2	>0.05
	Epirubicin	12	27.3	0.026
Anti HER2 agents	Trastuzumab Monotherapy	39	88.6	>0.05
	Dual therapy (Trastuzumab + Pertuzumab)	5	11.4	>0.05
Age (mean)	50.4 ± 8			>0.05
BSA (mean)	1.6 ± 0.17 m^2^			> 0.05

ER, estrogen receptors; PR, progesterone receptors; BSA, body surface area.

### Cardiotoxicity and cardiovascular events

Nineteen patients (43.2%) experienced at least one cardiac event: 11 patients (25%) developed cardiotoxicity (five of these patients developed cardiotoxicity after another cardiac event), and 13 patients (29.5%) had at least one other cardiac event (8 of these developed at least one cardiac event other than cardiotoxicity; Table 2, Fig. 1). Anginal symptoms showed marginal significance in stratifying the risk of cardiotoxicity after treatment.

**Table 2:** Cardiac events occurred throughout the study

Cardiac complications and cardiotoxicity	Num	%	*P*value of cardiotoxicity development risk
Need to temporary pause of anti-HER2 treatment	3	6.8	>0.05
Cardiac events	19	43.2	-
Cardiac event other than cardiotoxicity	13	29.5	-
Cardiac events have progressed to cardiomyopathy	5	11.4	-
Treatable palpitation non-AF	5	11.4	>0.05
New onset atrial fibrillation	3	6.8	>0.05
New onset of anginal symptoms	10	22.7	0.047
New diagnosed hypertension	3	6.8	>0.05
Cardiotoxicity according to the definitions described above	11	25	-
EF decreased ≥10% below 50%	7	15.9	-
EF decreased ≥15%	4	9	-

AF, atrial fibrillation; EF, ejection fraction.

### LVEF changes during study phases

**Figure 2.**
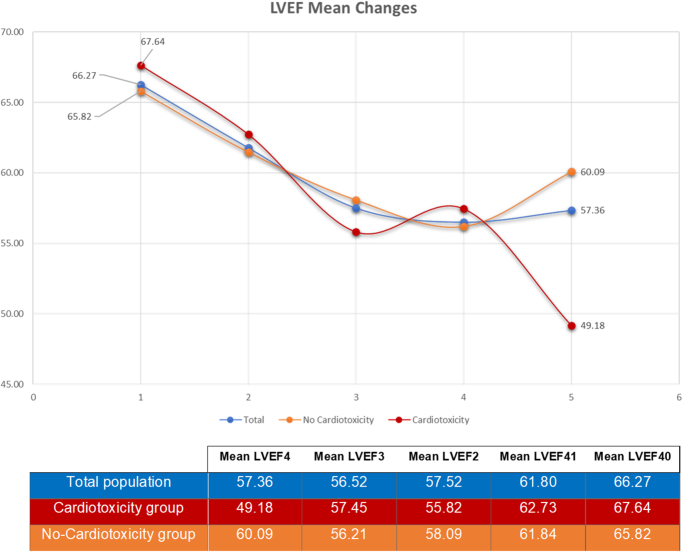
Left ventricular ejection fraction (LVEF) mean changes during the study phases.

The average decline in LVEF differed significantly across study phases. In the cardiotoxic group, LVEF decreased from 68% at baseline to 49% at the end of the study. Similar significant declines were observed in the non-cardiotoxic group and the total population (Fig. 2). While mean LVEF differences among most phases were significant, neither these differences (Supplemental Digital Content Table 1, available at: http://links.lww.com/MS9/B5), nor the degree of decline (Supplemental Digital Content Table 2, available at: http://links.lww.com/MS9/B5), nor the LVEF value at phase 1 or 2 (Supplemental Digital Content Table 3, available at: http://links.lww.com/MS9/B5) correlated with the risk of cardiotoxicity.

### Biomarker profiles and association with cardiotoxicity risk

Hs-Tn I concentrations rose significantly during treatment (Table 3). While no baseline hs-Tn I exceeded 40 ng/L, an increased number of patients surpassed this threshold at phase 1 and subsequently at phase 2 (Table 4).

**Table 3:** Biomarkers mean ± standard deviation measurements

		Phase 0	Phase 1	Phase 2
hs-CRP mg/L	Mean ± SD	**1.69 ± 2.4**	**4 ± 3.15**	**7.34 ± 4.46**
	P value of Mean differences	< 0.001		
			< 0.001	
	Min	0.14	1.3	1.6
	Max	11.4	14.2	16.6
IL-6 pg/mL	Mean ± SD	**2.21 ± 0.63**	**6.14 ± 6.63**	**15.64 ± 29.53**
	*P* value of mean differences	<0.001		
			<0.01	
	Min	1.13	1.78	2.17
	Max	3.86	34.7	146.9
NTproBNP pg/mL	Mean ± SD	**25.15 ± 12.9**	**30.84 ± 18.42**	**83.75 ± 50.93**
	*P* value of mean differences	>0.05		
			< 0.001	
	Min	8.5	10	13.3
	Max	65.8	80.9	213.5
U.A. mg/dL	Mean ± SD	**3.63 ± 0.93**	**4.63 ± 1.26**	**5.81 ± 1.46**
	*P* value of mean differences	<0.001		
			<0.001	
	Min	2.7	2.9	3.5
	Max	5.9	6.72	8.3
hs-Tn I ng/L	Mean ± SD	**14.68 ± 5.98**	**37.9 ± 20.61**	**66.43 ± 22.05**
	*P* value of mean differences	<0.001		
			<0.001	
	Min	9.3	9.5	27.10
	Max	27.8	90.3	90.6

Hs-CRP, high-sensitivity C reactive protein; Hs-Tn I, high-sensitivity troponin I; IL-6, interleukin 6; max, maximum value; min, minimum value; NTproBNP, N-terminal pro b-type natriuretic peptide; SD, standard deviation; U.A., uric acid.

**Table 4:** Biomarkers values above the standard cutoff in each phase

	Phase 0		Phase 1		Phase 2	
	Num	%	Num	%	Num	%
hs-CRP > 3 mg/L	5	11.4	18	40.9	40	90.9
IL-6 > 3 pg/mL	5	11.4	31	70.5	43	97.7
NTproBNP > 125 pg/mL	0	0	0	0	6	13.6
U.A. > 5.5 mg/dL	6	13.6	19	43.2	26	59.1
Hs-Tn I > 40 ng/L	0	0	14	31.8	34	77.3

Hs-CRP, high-sensitivity C reactive protein; Hs-Tn I, high-sensitivity troponin I; IL-6, interleukin 6; max, maximum value; min, minimum value; NTproBNP, N-terminal pro b-type natriuretic peptide; U.A., uric acid.

Hs-CRP levels also increased significantly (Table 3). While only five patients (11.4%) had baselines hs-CRP >3 mg/L (cardiovascular risk cutoff), an increased number of patients surpassed this threshold at phase 1 and subsequently at phase 2 (Table 4).

At phase 0, only hs-CRP showed significant predictive value for the risk of cardiotoxicity. However, both hs-CRP and hs-Tn l at phase 1 and phase 2 were strongly associated with the risk of cardiotoxicity (Table 5).

**Table 5:** Logistic regression results of the prediction of biomarkers for cardiotoxicity development risk

**A- Biomarkers predictive value for cardiotoxicity risk at phase 0**				
Phase 0		***P* value**		**Exp(B)**
hs-CRP mg/L		0.028		2.325
IL-6 pg/mL		0.084		
U.A. mg/dL		0.652		
hs-Tn I ng/L		0.530		
NTproBNP pg/mL		-		
**B- Biomarkers predictive value for cardiotoxicity risk at phase 1**				
Phase 1		***P* value**		**Exp(B)**
hs-CRP mg/L		0.012		1.737
IL-6 pg/mL		0.574		
U.A. mg/dL		0.693		
hs-Tn I ng/L		0.008		1.056
NTproBNP pg/mL		0.064		
	***P* value**	**Exp(B)**		**CI 95%**
hs-CRP mg/L	0.012	1.605		1.109–2.234
hs-Tn I ng/L	0.021	1.06		1–1.114
**C- Biomarkers predictive value for cardiotoxicity risk at phase 2**				
Phase 2		***P* value**		**Exp(B)**
hs-CRP mg/L		0.036		1.182
IL-6 pg/mL				
U.A. mg/dL				
hs-Tn I ng/L		0.005		1.108
NTproBNP pg/mL		0.036		0.977
	***P* value**	**Exp(B)**		**CI 95%**
hs-CRP mg/L	0.026	1.436		1.045–1.975
hs-Tn I ng/L	0.043	1.144		1.004–1.302
NTproBNP pg/mL	0.204	0.984		-
	***P* value**	**Exp(B)**	**CI 95%**	
hs-CRP mg/L	0.034	1.356	1.023–1.797	
hs-Tn I ng/L	0.013	1.171	1.034–1.326	

Hs-CRP, high-sensitivity C reactive protein; Hs-Tn I, high-sensitivity troponin I; IL-6, interleukin 6; max, maximum value; min, minimum value; NTproBNP, N-terminal pro b-type natriuretic peptide; U.A., uric acid.

None of the patients had an NTproBNP value above the standard cutoff (125 pg/mL) at either baseline or phase 1 (Table 4). Neither NTproBNP, IL-6, nor U.A. levels demonstrated a significant association with the risk of cardiotoxicity (Table 5), despite significant mean differences across phases (Table 3).

### Biomarker cutoffs and risk stratification

**Figure 3.**
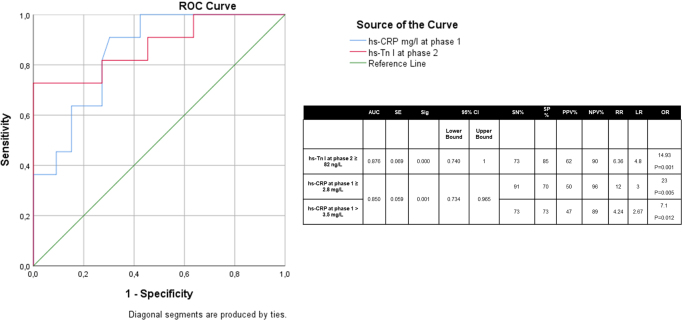
ROC curves of hs-Tn I after four cycles of anti-HER2 agents, and hs-CRP after four cycles of anthracyclines, for cardiotoxicity development risk. AUC, area under the curve; CI, confidence interval; Hs-CRP, high-sensitivity C reactive protein; Hs-Tn I, high-sensitivity troponin I; SE, standard error; Sig, significance (*P*-value); SN, sensitivity; SP, specificity; PPV, positive predictive value; NPV, negative predictive value; RR, relative risk; LR, likelihood ratio; OR: odds ratio.

We identified hs-Tn I values ≥82 ng/L after four cycles of anti-HER2 therapy as a critical threshold for high risk of cardiotoxicity (Fig. 3). Similarly, for hs-CRP, we found that values ≥2.8 mg/L after 4 cycles of ANT could play an important predictive role regarding the high risk of cardiotoxicity (Fig. 3). Another probable cutoff (hs-CRP > 3.5 mg/L) could also be considered (Fig. 3). Other possible cutoffs across the other phases are shown in Supplemental Digital Content Table 4, available at: http://links.lww.com/MS9/B5.

**Figure 4.**
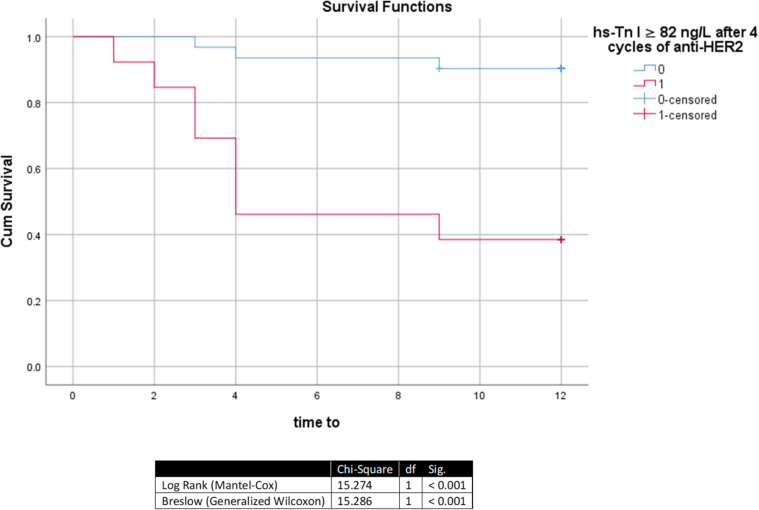
Kaplan–Meier analysis of developing cardiotoxicity in patients with hs-Tn I ≥ 82 ng/L after 4 cycles of anti-HER2 agents regardless of hs-CRP. Df, degrees of freedom; Hs-Tn I, high-sensitivity troponin I; Sig, Significance (*P*-value).

**Figure 5.**
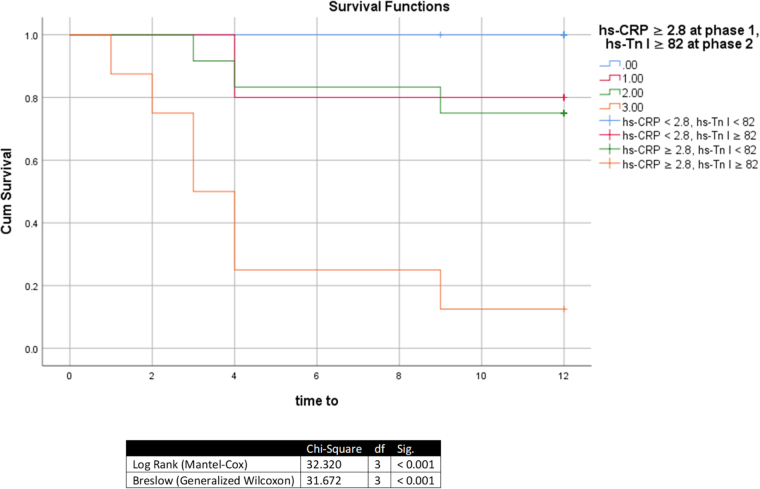
Kaplan–Meier analysis of developing cardiotoxicity in patients with hs-Tn I ≥82 ng/L after four cycles of anti-HER2 agents and hs-CRP ≥2.8 mg/L after four cycles of anthracyclines. df, degrees of freedom; Hs-CRP, high-sensitivity C reactive protein; Hs-Tn I, high-sensitivity troponin I; Sig, significance (*P*-value).

Kaplan–Meier analysis indicated that the highest-risk period within the first year of treatment was the first 4 months. Approximately 55% of patients with hs-Tn I ≥82 ng/L developed cardiotoxicity within this period (Fig. 4), and 75% of patients with both hs-Tn I ≥82 ng/L after four cycles of anti-HER2 therapy and hs-CRP ≥2.8 mg/L after 4 cycles of ANT developed cardiotoxicity within the same period (Fig. 5).

However, analyzing the role of hs-CRP and hs-Tn I across all phases using Bonferroni Correction and Cox regression revealed that for hs-CRP values, the level at phase 1 was the most significant predictor of cardiovascular risk development. For hs-Tn I, the value at phase 2 was a more significant predictor of cardiovascular risk development than the value at phase 1.

Cox regression analysis revealed that the most important interaction between biomarkers was between hs-CRP at phase 1 and hs-Tn I at phase 2. A significant interaction was also observed between baseline hs-CRP and hs-Tn I at phase 1 (Table 6). Additionally, the interaction between the two biomarkers within the same phase significantly increased the risk of cardiotoxicity (Supplemental Digital Content Table 5, available at: http://links.lww.com/MS9/B5).

**Table 6:** Cox regression analysis for prediction of cardiotoxicity risk within one-year post-treatment based on hs-CRP and hs-Tn I above the detected cutoffs

	SE	Sig	HR
hs-Tn I at phase 2 ≥ 82 ng/L	0.680	0.001	8.702
hs-CRP at phase 1 ≥ 2.8 mg/L	1.05	0.010	15.145
hs-CRP at phase 1 ≥ 2.8 mg/L AND hs-Tn I at phase 2 ≥ 82 ng/L	0.639	<0.001	14.186

### Treatment regimen and cardiotoxicity risk

Dual anti-HER2 agents did not influence the risk of cardiotoxicity. However, patients receiving epirubicin exhibited a 5.4-fold higher likelihood of developing cardiotoxicity compared to those receiving other ANT (*P* = 0.026; Table 1).

## Discussion

The use of anti-HER2 antibodies, especially TRAS, in breast cancer treatment has significantly improved disease outcomes, reducing recurrence by up to 40% and mortality by 34%^[9]^. Unexpectedly, however, this therapy has associated with cardiac injury, which was mostly asymptomatic^[10]^. Cardiac events following treatment, particularly when combined with ANT, raised concerns about the cardiac safety of this protocol. To date, no laboratory marker can predict this injury before clinical or imaging manifestations, despite ongoing research for specific/non-specific biomarkers. Key challenges remain selecting relevant biomarkers and determining optimal timing for predictive assessment during treatment.

In this study, cardiotoxicity occurred in 11 patients (25%) after treatment completion or during follow-up. This rate is moderately high. Notably, no cardiotoxicity developed directly after ANT or during the post-ANT treatment phase. Nevertheless, cardiotoxicity cannot be attributed solely to anti-HER2 agents; the role of ANT in its pathophysiology remains significant. In a previous similar study, we conducted on a different patient cohort (assessing only hs-Tn I), results aligned regarding injury timing post-treatment completion^[11]^.

Current LVEF measurement methods still lack sensitivity for detecting early-stage cardiotoxicity. Available imaging modalities cannot identify injury until weeks or months after onset, especially since patients may receive other therapies besides ANT and/or anti-HER2 agents and have variable cardiovascular risk factors. This necessitates cardio-oncology teams exploring alternative methods and biomarkers for early detection before LVEF decline. Thus, investigating cardiac biomarkers, particularly specific ones like troponin and natriuretic peptides, is valuable.

While cardiac troponin is primarily used to diagnose acute coronary events, it may play a vital role in detecting cancer-therapy-induced cardiac injury. Tn is part of the cardiac contractile apparatus. Tn I is more cardiac specific (vs. Tn T) for detecting myocardial injury^[12]^.

Clinical studies investigating troponin as a specific biomarker for ANT-induced cardiotoxicity show that its elevation is associated with a seven-fold increased risk of left ventricular dysfunction^[12]^. Its role in TRAS-related cardiotoxicity remains under investigation.

Since HER2-positive breast cancer treatment typically combines ANT and TRAS, pinpointing the exact cause of biomarker is challenging. This highlights the need for appropriate biomarker selection, optimal timing for testing, and robust monitoring strategies.

Elevated serum Tn levels after ANT completion but before starting TRAS may indicate higher cardiotoxicity risk^[13]^. Our current study supports this, showing statistically significant elevations in hs-Tn I at both phase1 and phase 2. Crucially, the clinical significance of hs-Tn I elevation after starting anti-HER2 therapy confirms that TRAS substantially exacerbates ANT-induced cardiac injury and accelerates clinical manifestation.

This aligns with our prior study on hs-Tn I predictive value in HER2-positive breast cancer^[11]^. Further supporting evidence comes from a cohort study of 12 500 breast cancer patients with 8-year follow-up: Among the 3.5% receiving combined ANT + TRAS therapy, higher cardiotoxicity rates were observed compared to ANT-alone or TRAS-alone groups. Notably, the TRAS-alone group still showed higher cardiotoxicity than ANT-alone group^[14]^.

Recent studies notably reveal that hs-Tn assays can detect minor myocardial injury (equivalent to 2–5 mg of myocardial cell mass), reflected by hs-Tn I changes of just 1–2 ng/L^[15]^. A >30% hs-Tn I increase (mean: 4 ng/L) between two measurements (same lab/assay) indicates cardiac tissue damage of ~10–20 mg – undetectable by current imaging modalities, even high-sensitivity ones^[16,17]^.

Optimal timing for hs-Tn I assessing is debated. Many studies assessed it hours/days post-ANT; others use weekly/monthly post-ANT monitoring^[18]^. While some studies linked Tn elevation during TRAS (+ANT) to subsequent LVEF decline, others report no statistically significant association between Tn rise and LVEF drop^[1,19]^.

In both our previous hs-Tn I study and current multi-biomarker study, we used identical sampling protocols. Our results align with Putt^[20]^, Ky^[21]^, and Sawaya^[22,23]^ regarding hs-Tn I predictive value for cardiotoxicity risk. However, Cardinale associated Tn I elevations more strongly with reversibility of TRAS-associated cardiac injury than with its initial development^[24]^.

Although NTproBNP changes between phase 1 and phase 2 were statistically significant in our study, it failed to predict cardiotoxicity risk in multivariate regression models. In univariate analysis, it showed statistical but no clinically relevant predictive value.

NTproBNP is released in response to myocardial wall stress, typically triggered by hemodynamic strain or structural necrosis, such as that induced by ANT chemotherapy^[25]^. In patients treated with ANT, direct cardiomyocyte injury via oxidative stress leads to rapid onset of wall stress, resulting in strong predictive performance of NTproBNP^[26]^. In contrast, TRAS exerts its cardiotoxic effects by disrupting ErbB2/ErbB4-mediated survival signaling, which compromises sarcomere integrity and calcium handling without causing immediate necrosis or hemodynamic overload^[27]^. Consequently, NTproBNP elevation in HER2+ patients typically occurs only after functional cardiac decline, such as reduced LVEF, limiting its utility as an early predictive biomarker.

Some studies showed that NTproBNP levels peak within 24–72 hours following ANT administration^[25,26]^. In contrast, NTproBNP elevation during TRAS therapy emerges more gradually, typically 3–6 months after initiation, coinciding with measurable LVEF decline^[28]^.

Of 16 studies testing NTproBNP, only 5 demonstrated a predictive value for TRAS-related cardiotoxicity risk^[25,29–32]^ (Supplemental Digital Content Table 6, available at: http://links.lww.com/MS9/B5). Several studies have explored this association, but results are inconsistent. Romano *et al* demonstrated that NTproBNP elevations during ANT therapy correlated with later cardiotoxicity within 3–12 months^[25]^, whereas their study and Zardavas *et al* also observed NTproBNP increases during TRAS treatment^[25,31]^. Contrarily, other investigations failed to confirm a predictive link^[8,20–23,28,33–37]^, possibly due to varied cardiotoxicity definitions, assay types, and sample sizes.

Several confounding factors complicate interpretation; diabetes mellitus increases cardiotoxic risk and impairs renal NTproBNP clearance^[28]^; prior ANT exposure is a significant predictor of subsequent TRAS-induced dysfunction, with early LVEF decline during ANT therapy^[18]^. Additionally, age-related threshold for NTproBNP lack standardization, with suggested cutoffs ranging from <125 pg/mL for individuals under 50 to <450 pg/mL for those over 75 years^[38]^.

Clinically, NTproBNP is limited as a standalone predictor due to its sensitivity to late-stage hemodynamic stress rather than early molecular or subcellular disruption^[39]^. It fails to detect early cardiomyocyte dysfunction such as ErbB2-PI3K/Akt pathway inhibition^[27]^, diastolic calcium leakage, and metabolic shifts^[11]^. Moreover, echocardiographic parameters like global longitudinal strain (GLS) reduction >15% offer earlier detection of myocardial injury than NTproBNP alone^[40]^.

Multi-marker strategies, particularly panels combing NTproBNP, yield enhanced diagnostic accuracy compared to dynamic NTproBNP monitoring alone. The most plausible explanation is that NTproBNP contributes to risk assessment only when combined with other biomarkers (hs-Tn I and hs-CRP), not independently. Future studies may establish new NTproBNP cutoffs distinct from chronic/acute heart failure references due to differing injury mechanisms.

hs-CRP, IL-6, and U.A. showed statistically significant changes across study phases. As ANT primarily triggers oxidative stress and inflammation, these inflammatory marker changes are expected.

However, only hs-CRP achieved statistical significance for predicting cardiotoxicity risk. IL-6 and U.A. failed to show predictive value. hs-CRP independently predicted cardiotoxicity risk at all study phases, most significantly post-ANT completion.

Our statistical analysis showed that baseline hs-CRP changes were statistically significant in predicting cardiotoxicity risk. ROC curve analysis identified two important baselines hs-CRP thresholds: >1.25 mg/L and >3 mg/L. However, only the first threshold (>1.25 mg/L) demonstrated good sensitivity and specificity for predicting cardiotoxicity development risk. Conversely, the time-dependent analysis of these values using Cox regression showed that clinical significance was most important for values exceeding 3 mg/L, while the risk actually begins at values above 1.25 mg/L. However, the highest clinical significance was observed for hs-CRP changes in phase 1, particularly at the ROC-derived threshold of ≥2.8 mg/L, which showed stronger predictive cutoff than the alternative one >3.5 mg/L. Notably, despite persistent statistical significance, clinical relevance (represented by Odds Ratio) decreased with higher phase 1 hs-CRP values and did not increase with elevated phase 2 levels despite statistical significance. This indicates that hs-CRP primarily signals the inflammatory threshold triggering subclinical cardiac injury, while subsequent injury progression, though linked to initial or ongoing inflammation, follows a new trajectory influenced by multiple factors including primary inflammation (cancer, or ANT-induced), anti-HER2 agents, and potential intrinsic cardiac factors. Supporting this, Cox regression analysis of biomarker interactions showed the strongest interaction between phase 1 hs-CRP (above studies thresholds) and phase 2 hs-Tn I, followed by baseline hs-CRP (>3 mg/L) with phase 1 hs-Tn I, both more significant than same-phase interactions. This confirms the greater impact of baseline inflammation preceding cardiac injury compared to concurrent inflammation. Consequently, the clinically critical change in hs-CRP is its elevation beyond a specific threshold regardless of magnitude, contrasting with hs-Tn I where higher levels reflect greater injury severity and cardiotoxicity probability. Persistent inflammation releases additional hs-CRP and other inflammatory markers, explaining their statistically significant phase-to-phase differences but a lack of independent predictive value for cardiotoxicity risk during established subclinical injury, where hs-Tn I dominates risk prediction.

From one perspective, the exacerbation of ANT-induced cardiac injury following TRAS administration can be explained through the vital biological role of NRG1/ErbB signaling pathways. These pathways serve as compensatory mechanisms under physiological and pathological stress, including exposure to cardiotoxic agents like ANT. TRAS inhibits NRG1-induced ErbB4/ErbB heterodimer formation, explaining both TRAS-associated cardiotoxicity and its synergistic toxicity with ANT. ANT exposure upregulates ErbB2 receptor expression in cardiomyocytes as a compensatory mechanism to activate protective intracellular pathways promoting proliferation and repair. However, TRAS-mediated receptor blockade impairs cardiac adaptation to ANT-induced oxidative stress, exacerbating ANT’s oxidative and inflammatory injury^[41,42]^.

From another perspective, ANT chemotherapy induces early oxidative and microvascular stress in the myocardium, disrupting mitochondrial function and endothelial integrity. These changes activate innate inflammatory pathways and generate a systemic inflammatory response detectable by hs-CRP. Thus, elevated hs-CRP after ANT plausibly reflects an inflammatory milieu and heightened cardiac vulnerability rather than immediate necrosis^[43,44]^.

This inflammatory priming reduces the heart’s adaptive reserve. When anti-HER2 therapies are introduced, they inhibit survival signaling (notably NRG1/ErbB-PI3K/Akt), which normally supports mitochondrial balance and stress adaptation in cardiomyocytes. In a pre-inflamed, metabolically fragile myocardium, HER2 blockade more readily tips cells toward injury, leading to membrane disruption and release of troponin. Clinically, hs-Tn I rise during anti-HER2 therapy, and prior ANT exposure is an independent risk factor for cardiotoxicity in this setting^[45–47]^.

The temporal pattern of biomarker changes supports a sequential “two hit” process: inflammation first (hs-CRP) followed by detectable injury (hs-Tn I). Imaging aligns with this sequence; GLS often deteriorates before LVEF declines and may accompany inflammatory signaling early on, whereas hs-Tn I elevation more closely parallels injury during anti-HER2 treatment. Together, serial hs-Tn I and GLS improve early detection and tracking of cancer therapy-related cardiac dysfunction^[46–48]^; however, we could not perform the GLS in our study.

Practically, an elevated hs-CRP at the end of ANT therapy can flag patients for closer surveillance when stating anti-HER2 agents, using serial hs-Tn I and GLS, if it is possible, to detect subclinical injury and guide timely adjustments. Tight control of cardiovascular risk factors and consideration of cardioprotective strategies could be discussed to mitigate ANT/HER2-related cardiotoxicity and preserve cardiac function.

Three major studies investigated hs-CRP as a biomarker for cardiotoxicity risk. Ky *et al* and Putt *et al* both reported significant hs-CRP elevation 3 months post-ANT initiation but found no predictive value for cardiotoxicity^[20,21]^.

Conversely, Onitilo *et al* demonstrated that hs-CRP >3 mg/L had high sensitivity for left ventricular dysfunction within subsequent 3 months^[49]^. Nevertheless, hs-CRP remains a sensitive but non-specific cardiac injury marker unsuitable for standalone risk assessment.

Our survival analysis identified the first 4 months post-treatment as the highest-risk period for cardiotoxicity during the 1-year follow-up. Patients with both post-TRAS hs-Tn I ≥82 ng/L and post-ANT hs-CRP ≥2.8 mg/L had the highest incidence (75%). For isolated hs-Tn I ≥82 ng/L (regardless of hs-CRP), 55% developed cardiotoxicity within this window.

Cardiovascular complications (primarily anginal symptoms) occurred in a significant proportion during active treatment but not post-therapy, suggesting transient drug-related injury. Oxidative stress directly affects cardiac automaticity and conduction by targeting ion channels (particularly potassium), causing ion action potential propagation abnormalities and arrhythmias^[50]^. Cellular studies show angiotensin II and phenylephrine suppress NRG1 production, while endothelial cells adjust NRG1 synthesis in response to circulating angiotensin II and epinephrine^[3]^.

Contrary to studies reporting lower cardiotoxicity with epirubicin versus doxorubicin, our epirubicin-treated patients showed higher risk – though larger samples are needed for confirmation. This contrasts with our prior study where dual HER2 blockade (TRAS + PERT) increased cardiotoxicity versus TRAS alone, whereas our current data align with CLEOPATRA^[51]^ showing no added risk from PERT. Li Zhang *et al* observed greater hs-Tn I elevations with dual HER2 blockade without increased clinical cardiotoxicity^[46]^. These observations warrant further study with larger cohorts and dedicated PERT-ANT investigations.

## Study limitations

The study faces several limitations: First, the relatively small sample size may limit the statistical significance of our analytical findings. Second, diagnosing cardiotoxicity via LVEF reduction relied solely on 2D echocardiography – a double-edged approach. While more precise modalities – particularly GLS, which is currently considered more accurate and sensitive than conventional LVEF measurements – were unavailable due to limited accessibility and high costs, this method offers practical advantages for resource-limited centers by providing an accessible, low-cost, and credible diagnostic tool. Finally, the follow-up duration (1-year post-treatment) remains shorter than the multi-year periods in major global studies.

## Conclusion

Cancer-therapy-associated cardiotoxicity remains a critical clinical complication, significantly impacting both oncological outcomes and the development of new cardiovascular pathologies. This dual effect influences prognoses for both conditions and ultimately patients’ quality of life. There is an urgent need to identify biomarkers predicting cardiotoxicity before clinical manifestation, enabling optimal management without compromising treatment efficacy or quality of life. Cardiac-specific biomarkers (e.g., troponin) and inflammatory markers (e.g., CRP) show promising potential in this regard. Further research with larger cohorts, standardized multi-assay approaches, and optimized sampling timelines is crucial to validate these findings and establish reliable predictive biomarkers for cancer-therapy-induced cardiotoxicity.

## Data Availability

The data that support the findings of this study are available on request from the corresponding author.

## Research registration unique identifying number (UIN)

Tishreen University Hospital issued approval 2022-CD-ON-64.
